# Serum Macro- and Trace-Element Alterations and Redox Imbalance in Cattle with Naturally Occurring Dermatophytosis

**DOI:** 10.3390/ani16131984

**Published:** 2026-06-27

**Authors:** Yusuf Umut Batı, Ali Haydar Kırmızıgül, Bengü Bilgiç, Fatih Büyük, Duygu Tarhan, Mert Sezer, Enes Akyüz, İbrahim Ertuğrul Yalçın, Tahir Gezer, Halil İbrahim Dilber, Yaren Ersoy, Gürbüz Gökce, Lale Başer, Mehmet Erman Or

**Affiliations:** 1 Department of Internal Medicine, Faculty of Veterinary Medicine, Kafkas University, Kars 36300, Türkiye; ahkirmizigul@kafkas.edu.tr (A.H.K.); mertsezer90@windowslive.com (M.S.); enesakyuz_44@hotmail.com (E.A.); dr.tahir36@hotmail.com (T.G.); ibrahim.dilber36@gmail.com (H.İ.D.); dr-gkce@hotmail.com (G.G.); 2 Department of Internal Medicine, Faculty of Veterinary Medicine, Istanbul University-Cerrahpaşa, Istanbul 34320, Türkiye; bengu.bilgic@iuc.edu.tr (B.B.); ermanor@iuc.edu.tr (M.E.O.); 3 Department of Microbiology, Faculty of Veterinary Medicine, Kafkas University, Kars 36100, Türkiye; fatihbuyuk36@gmail.com (F.B.); yarenbvet@gmail.com (Y.E.); 4 Department of Biophysics, School of Medicine, Bahçeşehir University, Istanbul 34734, Türkiye; duygu.tarhan@bau.edu.tr; 5 Department of Civil Engineering, Faculty of Engineering and Natural Sciences, Bahçeşehir University, Istanbul 34353, Türkiye; ibrahimertugrul.yalcin@bau.edu.tr; 6 Department of Biochemistry, Faculty of Veterinary Medicine, Kafkas University, Kars 36100, Türkiye; lalebesli10@gmail.com

**Keywords:** dermatophytosis, trace elements, oxidative stress

## Abstract

This study determined the etiological distribution of naturally occurring bovine dermatophytosis and evaluated associated serum macro- and trace-element and redox alterations. Seventy-five calves and young cattle aged 2–12 months, of both sexes and mixed local, Simmental, Brown Swiss/Montofon, and crossbred types, were included: 50 clinically and mycologically confirmed dermatophytosis cases and 25 clinically healthy controls. Skin scrapings and hair samples from affected cattle were examined by direct microscopy and culture. Single jugular blood samples were collected from all animals during the same seasonal period (February–May 2026), and serum mineral and redox markers were analyzed. Copper, zinc, cobalt, selenium, molybdenum, manganese, magnesium, calcium, sodium, iron, and chromium concentrations, catalase (CAT), reduced glutathione (GSH), and malondialdehyde (MDA) were measured. Data were analyzed using a general linear model including group and sex as fixed effects and age in months as a covariate. Model-adjusted results were expressed as least-squares means ± standard error (LSM ± SE). *Trichophyton verrucosum* and *T. mentagrophytes* were identified in 90% and 10% of affected cattle, respectively. Affected cattle had lower Cu, Zn, Se, Na, Fe (*p* < 0.01), Mg (*p* = 0.009), Ca (*p* < 0.001), CAT, and GSH (*p* < 0.001), but higher Co, Cr (*p* < 0.01) and MDA (*p* < 0.001). These findings suggest field associations between bovine dermatophytosis, mineral imbalance, and redox disturbance.

## Simple Summary

Bovine dermatophytosis, also known as ringworm, is a fungal skin disease of cattle. Its contagiousness and zoonotic relevance depend on the dermatophyte species involved; zoophilic species such as *Trichophyton verrucosum* and *Trichophyton mentagrophytes* may spread among animals and may also infect humans. This study examined cattle with naturally occurring ringworm to identify the fungi involved and to determine whether affected animals showed changes in blood mineral levels and in the balance between harmful oxygen-derived molecules and the body’s natural protective systems. The main fungus detected was *Trichophyton verrucosum*, while *Trichophyton mentagrophytes* was found less frequently. Cattle with ringworm had lower blood levels of several important minerals, including copper, zinc, selenium, magnesium, calcium, sodium, and iron, whereas cobalt and chromium levels were higher. They also showed weaker natural protection against harmful molecules and greater signs of cell damage. These findings suggest that ringworm in cattle may be associated with body-wide biochemical changes, not only visible skin lesions. Understanding these changes may help improve clinical assessment and herd health management.

## 1. Introduction

Dermatophytosis is a fungal infection of keratinized tissues caused by dermatophytes and is widely recognized as an important component of the “One Health” concept in both veterinary and human medicine [1,2,3]. However, its contagiousness and zoonotic relevance depend on the dermatophyte species involved. In cattle, *Trichophyton verrucosum* is recognized as the principal causative agent worldwide and is considered an important zoophilic and zoonotic dermatophyte [4]. Other zoonotic dermatophytes, including *Trichophyton mentagrophytes* and *Microsporum canis*, may also be relevant in animal and human dermatophytosis, although their occurrence varies according to host species, geography, and epidemiological context [2,5,6,7]. In contrast, *Microsporum gypseum* is a geophilic species primarily associated with soil-related environmental exposure and should not be interpreted as a typical agent of contagious transmission. As one of the most common dermatological diseases in cattle worldwide, bovine dermatophytosis remains a significant threat to herd health and productivity, particularly in intensive production systems, where it may become endemic [4,8]. This pattern is also consistent with previous regional observations indicating the frequent involvement of *T. verrucosum* in bovine dermatophytosis cases [9]. The persistence of dermatophyte arthroconidia in contaminated housing environments, equipment, and fomites may complicate control measures and contribute to recurrent outbreaks, particularly when host-adapted or zoophilic dermatophytes are involved [10,11].

The clinical presentation of bovine dermatophytosis is influenced by the interaction between fungal virulence and the host immune response. Typical lesions are characterized by alopecic and hyperkeratotic areas that tend to expand peripherally, with a scaly center and gray-white, asbestos-like crusts [12,13]. Although the lesions are most commonly localized to the head, neck, periorbital region, and rump, generalized forms may also occur [6,7,14]. Clinical manifestations such as restlessness, pruritus, and stress in infected animals may contribute to reduced feed intake and stress-related metabolic responses [15].

Macro and trace elements play vital roles in maintaining the overall health of cattle and supporting their resistance to infectious diseases. From a nutritional physiology perspective, these elements function as cofactors in the formation of cellular structural components, enzyme activation, hormone synthesis, and, in particular, the regulation of immune function [16]. In the pathogenesis of dermatophytosis, alterations in macro- and trace-element status may be associated with oxidative stress and antioxidant defense. In this context, copper (Cu), zinc (Zn), cobalt (Co), selenium (Se), molybdenum (Mo), manganese (Mn), magnesium (Mg), calcium (Ca), sodium (Na), iron (Fe), and chromium (Cr) were evaluated because of their biological relevance to mineral homeostasis, immune function, and redox balance [16,17].

Among these elements, Zn is of particular importance because of its critical role in epithelial regeneration and keratinization. Zinc supports cell proliferation by serving as a structural and functional component of numerous enzymes, including DNA and RNA polymerases. In cases of zinc deficiency, parakeratosis and hyperkeratosis may develop, thereby weakening the skin barrier and facilitating invasion by *Trichophyton* spp. [16]. Similarly, Cu is essential for the activity of lysyl oxidase, which is involved in collagen and elastin synthesis, as well as superoxide dismutase (SOD), an important antioxidant enzyme. Copper deficiency not only impairs skin pigmentation but also reduces the phagocytic capacity of neutrophils and macrophages, thereby increasing susceptibility to infection [18].

Selenium, as an essential component of glutathione peroxidase (GSH-Px), helps neutralize excess free radicals generated during infection and thereby limits cellular damage [19]. Fe is an essential trace element for erythropoiesis and also plays an important role in the growth, differentiation, and function of immune cells [20,21]. Co is essential for vitamin B12 synthesis by rumen microorganisms and therefore indirectly supports energy metabolism and erythropoiesis [22]. Cr has been associated with stress-related metabolic and immune regulation in cattle, although responses may vary depending on physiological and experimental conditions [17]. In addition to trace elements, Ca, Mg, and Na are involved in neuromuscular function, membrane stability, osmotic balance, acid–base regulation, and several enzymatic processes; therefore, their assessment may help characterize broader mineral alterations in cattle with dermatophytosis [16].

Reactive oxygen species (ROS), which may increase during the inflammatory processes associated with dermatophyte infection, can induce oxidative stress and cause cellular damage to proteins, lipids, and nucleic acids [23,24]. Catalase (CAT), one of the major enzymatic antioxidants, protects biomolecules against oxidative damage by catalyzing the conversion of hydrogen peroxide into water and oxygen [24]. Reduced glutathione (GSH), in contrast, is a major intracellular non-enzymatic antioxidant that plays a central role in the detoxification of free radicals and other pro-oxidant compounds, as well as in the maintenance of cellular redox homeostasis [25]. Malondialdehyde (MDA), on the other hand, is regarded as one of the major end products of lipid peroxidation, and increased levels are considered a biochemical indicator of oxidative stress-related lipid damage [26]. Indeed, a recent study on bovine trichophytosis reported increased MDA levels together with decreased CAT and other antioxidant defense markers in infected animals, indicating that the disease is not merely a localized skin infection but may also disrupt systemic oxidative balance [27].

Although a limited number of studies have investigated mineral parameters such as zinc and copper in relation to bovine dermatophytosis [28], research evaluating macro- and trace-element profiles together with oxidative stress and antioxidant defense markers remains limited. Recent studies conducted in cattle under different metabolic conditions have shown that the integrated assessment of serum macrominerals, trace elements, and oxidative status indicators provides a valuable approach to understanding systemic disease processes [29]. Macro and trace elements, including Cu, Zn, Co, Se, Mo, Mn, Mg, Ca, Na, Fe, and Cr, play important biological roles in maintaining skin integrity, supporting epithelial regeneration, preserving immune function, and sustaining the metabolic balance associated with infection. Likewise, CAT, reduced GSH, and MDA are considered valuable biochemical markers for assessing oxidative stress and antioxidant defense capacity during the course of infection. In this context, the present study aimed to comparatively evaluate serum Cu, Zn, Co, Se, Mo, Mn, Mg, Ca, Na, Fe, and Cr concentrations together with CAT, GSH, and MDA levels in healthy cattle and cattle with dermatophytosis, and to assess the associations of naturally occurring dermatophytosis with macro- and trace-element profiles and oxidative balance under field conditions.

## 2. Materials and Methods

### 2.1. Ethical Approval

The present study was approved by the Local Ethics Committee for Animal Experiments of Kafkas University (decision no. KAU-HADYEK/2026-032). Blood samples were collected in accordance with standard procedures, and all reasonable measures were taken to minimize stress and avoid harm to the animals. All procedures involving animals were performed in accordance with institutional guidelines and national regulations for animal welfare.

### 2.2. Animal Material

**Figure 1:**
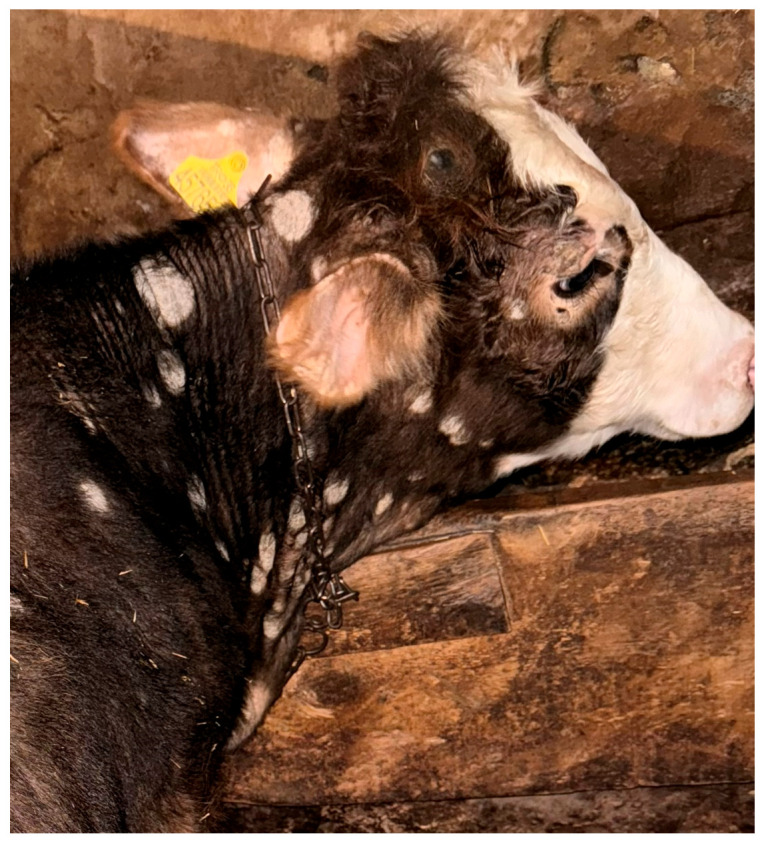
Clinical appearance of a cow with dermatophytosis included in the study.

A total of 75 cattle aged 2 to 12 months were included in the study without breed- or sex-based selection. Group allocation was based on laboratory diagnostic findings together with clinical evaluation. The dermatophytosis group comprised 50 cattle with clinical lesions compatible with dermatophytosis affecting various parts of the body (Figure 1). In these animals, dermatophyte structures were detected by direct microscopic examination of skin scrapings and hair samples collected from the affected areas.

The control group consisted of 25 clinically healthy cattle from different farms that were raised under broadly similar regional management, housing, and feeding conditions to those of the affected group and showed no skin lesions or clinical signs of systemic disease on examination. In the study region, winter feeding is commonly based on summer-harvested meadow hay offered together with straw; however, because the animals originated from different farms, possible variations in pasture origin, hay quality, straw quality, and farm-level feeding practices could not be completely excluded.

The study population consisted of calves and young stock. Adult lactating cows, pregnant cows, feedlot-finishing steers, and mature breeding bulls were not included. The dermatophytosis group included 25 males and 25 females, whereas the control group included 14 males and 11 females. The animals represented the mixed young cattle population commonly raised under local field conditions. The dermatophytosis group included local cattle (*n* = 18), Simmental (*n* = 13), Simmental crossbreds (*n* = 11), Brown Swiss/Montofon (*n* = 2), Brown Swiss/Montofon crossbreds (*n* = 4), and local × Simmental crossbreds (*n* = 2). The control group included local cattle (*n* = 13), Simmental (*n* = 4), Simmental crossbreds (*n* = 3), Brown Swiss/Montofon (*n* = 2), Brown Swiss/Montofon crossbreds (*n* = 2), and local × Simmental crossbreds (*n* = 1). Animals were not stratified according to breed type or narrower age subgroups, and these variables were not used as grouping criteria. Sex was recorded descriptively and subsequently included as a fixed effect in the statistical model. The control animals were selected from the same geographical region and broadly similar field management conditions, but they were not individually matched with affected cattle at the farm level. Therefore, equal numbers of dermatophytosis and control animals from each farm could not be achieved under field conditions, and farm-level variation was considered a potential source of variability.

Clinical lesion distribution was recorded during examination; however, a predefined published or validated dermatophytosis severity scoring system was not applied prospectively. Therefore, disease severity was not used as a stratification variable in the present study.

### 2.3. Mycological Analysis

Before sampling, the skin lesions of cattle with clinical signs of dermatophytosis were cleaned with cotton soaked in 70% alcohol. After evaporation of the alcohol, skin scrapings and hair samples were collected from the margins of the keratinized lesions into sterile plastic Petri dishes using a sterile scalpel. A portion of each sample was used for direct microscopic examination, and the remainder was used for culture analysis [9].

### 2.4. Direct Microscopic Examination

For direct microscopic examination, a portion of the collected skin scrapings and hair samples was placed on a clean glass slide with one drop of 10% potassium hydroxide (KOH) solution (Sigma-Aldrich, St. Louis, MO, USA; Cat. No. 417661). The preparation was then gently heated over a low Bunsen burner flame for approximately 1 min and covered with a coverslip. The prepared slides were subsequently examined under a light microscope at ×400 and ×1000 magnification. During examination, typical spores, arthrospores, and hyphal structures, as well as evidence of hair invasion by dermatophytes, were evaluated [9].

### 2.5. In Vitro Culture

For culture analysis, skin scrapings and hair samples were inoculated in duplicate onto slanted Dermatophyte Test Medium (D.T.M.) agar (HiMedia Laboratories Pvt. Ltd., Mumbai, India; M188) supplemented with Dermato supplement (HiMedia Laboratories Pvt. Ltd., Mumbai, India; FD015) to ensure selectivity. The samples were embedded in the agar surface. One set of slanted agar tubes was incubated at 25 °C and the other at 37 °C under aerobic conditions for 4 weeks. Fungal cultures were examined regularly at 4–5-day intervals throughout the incubation period. Species identification was based on published descriptions of cultural characteristics, colony morphology, and microscopic morphology after staining with lactophenol cotton blue (LPCB) (Sigma-Aldrich, St. Louis, MO, USA; Cat. No. 61335) [9,30,31].

### 2.6. Collection of Blood Samples

Blood samples were collected once from the jugular vein during the same seasonal study period for both the dermatophytosis and control groups. The samples were not collected on a single day; rather, sampling was performed on different days between February and May 2026 under field conditions. Sampling was carried out during routine daytime working hours, approximately between 09:00 and 17:00. Samples were collected before any antifungal treatment or mineral-related therapeutic intervention. Blood was obtained using an appropriate holder and a sterile collection needle (Vacuette^®^, Greiner Bio-One GmbH, Kremsmünster, Austria) and placed into serum tubes. To obtain serum, the samples were centrifuged at 3000 rpm for 10 min (Andreas Hettich GmbH & Co. KG, Tuttlingen, Germany). Serum samples for biochemical analyses were stored at −20 °C until analysis.

### 2.7. Measurement of Macro Minerals and Trace Elements

Bovine serum samples were transferred into sterile 2 mL Eppendorf tubes and transported to the laboratory under controlled conditions. All samples were stored at −20 °C until the day of analysis. On the day of digestion, the samples were allowed to equilibrate to room temperature, after which 0.500 mL aliquots were transferred into Teflon digestion vessels. For microwave-assisted digestion, 8 mL of concentrated nitric acid (65%, Merck KGaA, Darmstadt, Germany) was added to each sample. Digestion was performed using a Berghof MWS-2 microwave digestion system (Berghof Products + Instruments GmbH, Eningen unter Achalm, Germany) under controlled conditions to ensure complete mineralization of the organic matrix. Following digestion, the solutions were filtered through blue-band Whatman filter paper (Cytiva/Whatman, Marlborough, MA, USA) and quantitatively transferred into sterile 50 mL Falcon tubes (Corning Inc., Corning, NY, USA). The final volume was adjusted to 50 mL with ultrapure deionized water. Elemental concentrations of Cu, Zn, Co, Se, Mo, Mn, Mg, Ca, Na, Fe, and Cr were determined by inductively coupled plasma optical emission spectroscopy (ICP-OES; (PerkinElmer Inc., Waltham, MA, USA). Instrument calibration was performed using a certified multi-element standard solution (1000 mg/L, Merck KGaA, Darmstadt, Germany). Serum Na concentrations were expressed in µg/µL, whereas Cu, Zn, Co, Se, Mo, Mn, Mg, Ca, Fe, and Cr concentrations were expressed in µg/mL.

### 2.8. Measurement of Oxidative Stress and Antioxidant Parameters

Serum CAT activity, an important indicator of the enzymatic antioxidant defense system, was measured according to the method described by Aebi [32], based on monitoring the decrease in absorbance at 240 nm resulting from the enzymatic decomposition of hydrogen peroxide. The reduced GSH levels in the serum, one of the main components of the non-enzymatic antioxidant defense system, were determined according to the method described by Sedlak and Lindsay [33]. In this method, sulfhydryl groups in the sample react with 5,5′-dithiobis-(2-nitrobenzoic acid) (DTNB) to form a yellow-colored product, the absorbance of which was measured at 412 nm. Serum MDA levels, an important indicator of oxidative stress and one of the end products of lipid peroxidation, were determined by spectrophotometric measurement of the colored complex formed by the reaction of malondialdehyde with thiobarbituric acid at 532 nm [34]. All spectrophotometric measurements were performed using a microplate reader (Epoch, BioTek Instruments Inc., Winooski, VT, USA).

### 2.9. Statistical Analysis

Statistical analyses were performed using SPSS Statistics version 25.0 (IBM Corp., Armonk, NY, USA). A General Linear Model (GLM) procedure was applied to evaluate the effects of the study variables. Serum macro- and trace-element concentrations and oxidative stress parameters (CAT, GSH, and MDA) were included as dependent variables. Group and sex were included as independent variables (fixed effects), while age (in months) was included as a covariate. Statistical significance was set at *p* < 0.05. Quantitative data were expressed as Least Square Mean ± Standard Error (LSM ± SE). In addition, correlations between serum macro- and trace-element concentrations and oxidative stress/antioxidant parameters (CAT, GSH, and MDA) were evaluated separately in the dermatophytosis and control groups using Spearman’s correlation analysis.

## 3. Results

### 3.1. Clinical Findings

During the systemic clinical examination of the 50 cattle in the dermatophytosis group, in which infection was confirmed by mycological analysis, lesions were observed predominantly on the head, neck, periorbital region, and rump. The lesions exhibited a typical ringworm appearance, characterized by thick, gray-white, asbestos-like crusts, circular areas of alopecia, and marked desquamation.

### 3.2. Mycological Findings

**Figure 2:**
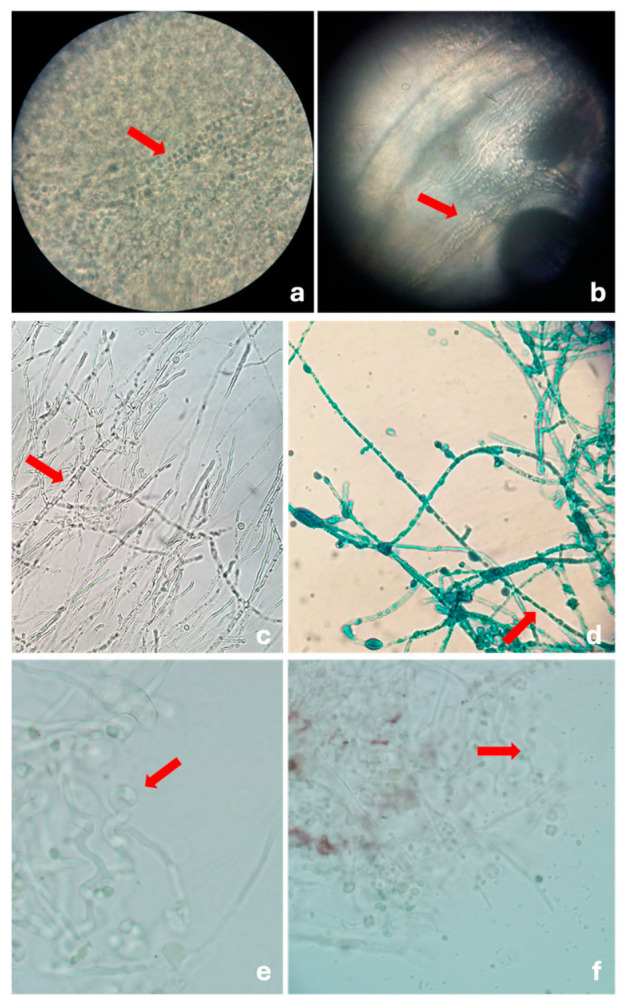
Direct microscopic findings of skin scrapings obtained from cattle with dermatophytosis following 10% KOH preparation, together with light micrographs of culture-positive isolates grown on D.T.M. agar and stained with lactophenol cotton blue (×1000). (**a**) Arthrospores (red arrow); (**b**) dermatophyte invasion of the hair shaft (red arrow); (**c**,**d**) typical chlamydospore chains (“chains of pearls”) of *T. verrucosum* (red arrows); and (**e**,**f**) spiral hyphae of *T. mentagrophytes* (red arrows).

On direct microscopic examination of skin scrapings and hair samples, arthrospore chains, hyphal structures, and evidence of dermatophyte invasion of hair shafts were observed, consistent with dermatophyte infection (Figure 2a,b). On slanted Dermatophyte Test Medium (D.T.M.) agar, fungal colony growth accompanied by a color change from yellow to pink or red, associated with the alkaline reaction developing during incubation at 25 °C and 37 °C, was considered indicative of dermatophyte growth. Culture analysis of skin scrapings and hair samples yielded *Trichophyton verrucosum* in 90% of the samples and *Trichophyton mentagrophytes* in 10%. Species identification was based on published descriptions of cultural characteristics, colony morphology, and microscopic morphology in lactophenol cotton blue-stained preparations. Isolates that grew slowly at both 25 °C and 37 °C, produced slightly folded, heaped, glabrous gray or white colonies, and exhibited septate hyphae with typical chlamydospore chains (“chains of pearls”) were identified as *T. verrucosum* (Figure 2c,d). Isolates that grew at 25 °C produced a color change from pink to red on D.T.M. agar, formed white colonies with radial folds, and exhibited pyriform microconidia, and club-shaped macroconidia, and typical spiral hyphae were identified as *T. mentagrophytes* (Figure 2e,f). Overall, 45 isolates (90%) were identified as *T. verrucosum* and 5 isolates (10%) as *T. mentagrophytes* (Table 1).

### 3.3. Serum Macro- and Trace-Element Concentrations

Comparative serum macro- and trace-element concentrations in healthy control cattle (*n* = 25) and cattle with dermatophytosis (*n* = 50) are presented in Table 2. Serum Cu, Zn, Se, Mg, Ca, Na, and Fe concentrations were significantly lower in the dermatophytosis group than in the healthy control group (Cu, Zn, Se, Na, and Fe: *p* < 0.01; Mg: *p* = 0.009; Ca: *p* < 0.001). Conversely, serum Co and Cr concentrations were significantly higher in cattle with dermatophytosis than in healthy controls (*p* < 0.01). No statistically significant differences were detected between the groups in serum Mo (*p* = 0.500) or Mn (*p* = 0.638) concentrations.

### 3.4. Serum Oxidative Stress and Antioxidant Defense Parameters

Comparative serum oxidative stress and antioxidant defense parameters in healthy control cattle (*n* = 25) and cattle with dermatophytosis (*n* = 50) are presented in Table 3. Serum CAT activity and GSH levels were significantly lower in the dermatophytosis group than in the healthy control group (*p* < 0.001). Conversely, serum MDA levels were significantly higher in cattle with dermatophytosis than in healthy controls (*p* < 0.001). The LSM ± SE values in the healthy control and dermatophytosis groups were 18.90 ± 1.36 and 4.67 ± 0.74 for CAT, 1.71 ± 0.051 and 0.716 ± 0.036 for GSH, and 8.63 ± 2.43 and 38.98 ± 1.75 for MDA, respectively.

### 3.5. Correlation Analysis

In addition to between-group comparisons, correlations between serum macro- and trace-element concentrations and oxidative stress/antioxidant defense parameters were evaluated separately in the dermatophytosis and healthy control groups (Table 4 and Table 5). In the dermatophytosis group, CAT activity was positively correlated with Cu (r_s_ = 0.429, *p* < 0.01), Zn (r_s_ = 0.284, *p* < 0.05), Mn (r_s_ = 0.349, *p* < 0.05), Mg (r_s_ = 0.398, *p* < 0.01), Na (r_s_ = 0.450, *p* < 0.01), Fe (r_s_ = 0.359, *p* < 0.05), and Cr (r_s_ = 0.314, *p* < 0.05). In the same group, GSH levels were negatively correlated only with Co (r_s_ = −0.290, *p* < 0.05), whereas MDA levels were negatively correlated with Cu (r_s_ = −0.295, *p* < 0.05), Mo (r_s_ = −0.413, *p* < 0.01), Mn (r_s_ = −0.385, *p* < 0.01), Mg (r_s_ = −0.314, *p* < 0.05), Fe (r_s_ = −0.294, *p* < 0.05), and GSH (r_s_ = −0.305, *p* < 0.05). In the healthy control group, CAT activity was positively correlated only with Ca (r_s_ = 0.653, *p* < 0.01), while neither GSH nor MDA showed significant correlations with serum macro- or trace-element concentrations.

## 4. Discussion

In this study, the etiological distribution of bovine dermatophytosis was determined, and infection-associated alterations in serum macro- and trace-element concentrations and oxidative stress/antioxidant defense parameters were comparatively evaluated. The findings suggest that cutaneous infections caused by *Trichophyton* spp. are not limited to superficial dermatological involvement, but may also be associated with systemic biochemical alterations affecting mineral homeostasis and redox balance in the host.

Mycological analysis of samples obtained from cattle with clinical signs of dermatophytosis revealed that *Trichophyton verrucosum* was isolated from 90% of cases (45/50), whereas *Trichophyton mentagrophytes* was isolated from 10% (5/50). This predominance supports the view that *T. verrucosum* is the principal etiological agent of bovine dermatophytosis. The finding is also consistent with previous reports indicating that bovine dermatophytosis may become endemic under intensive housing conditions, particularly during the winter months, when indoor housing, increased stocking density, and frequent direct contact among animals facilitate transmission [2,5,35]. The predominance of *T. verrucosum* in the present study may therefore be related to its host-adapted nature and its efficient spread through close animal-to-animal contact under confined housing conditions. Intensive management systems and environmental factors have also been reported to contribute to the dissemination and persistence of infection [7,14]. By contrast, the lower frequency of *T. mentagrophytes* may reflect its more sporadic involvement in cattle, as this species is more commonly associated with rodent reservoirs and may be transmitted indirectly through contaminated feed or rodent activity in the farm environment [1,4,14]. Thus, the limited detection of *T. mentagrophytes* shown in this study may indicate that its adaptation to cattle is less pronounced than that of the more host-adapted *T. verrucosum*.

Although the animals in both groups were maintained under broadly comparable regional feeding conditions, possible dietary and environmental influences on serum mineral concentrations should be considered. In the study region, winter feeding is commonly based on summer-harvested meadow hay offered together with straw; however, pasture origin, hay quality, straw quality, mineral supplementation practices, and farm-level feeding management may vary between herds. Since serum macro- and trace-element concentrations are directly influenced by dietary mineral intake, forage quality, environmental mineral availability, gastrointestinal absorption, mineral interactions, and homeostatic regulatory mechanisms [16], some of the observed differences in Cu, Zn, Se, Mg, Ca, Na, Fe, Co, and Cr concentrations may have been related to dietary or environmental variation. Therefore, the mineral alterations detected in this study should be interpreted as field associations observed in cattle with dermatophytosis rather than as definitive evidence that dermatophytosis alone caused these changes.

Another factor that may have influenced the measured serum parameters is the clinical severity of dermatophytosis. More extensive dermatophyte lesions may be associated with a stronger cutaneous inflammatory response [23], greater discomfort or stress and altered feed intake [15], and increased oxidative burden [24]. These changes may, in turn, influence serum macro- and trace-element concentrations through altered mineral intake, absorption, utilization, and homeostatic regulation [16], as well as CAT, GSH, and MDA levels through changes in oxidative stress and antioxidant defense [24]. In the present study, lesion distribution was recorded during clinical examination; however, a predefined published or validated clinical severity scoring system was not applied prospectively, and lesion number or lesion extent was not recorded in a standardized quantitative manner for all affected animals. Therefore, the possible influence of infection severity on serum mineral concentrations and oxidative stress/antioxidant defense markers could not be evaluated reliably. This should be considered when interpreting the biochemical differences observed between cattle with dermatophytosis and healthy controls.

In the present study, serum Zn concentrations were significantly lower in cattle with dermatophytosis than in healthy controls (*p* < 0.01), suggesting that trace-element homeostasis is altered during the disease and that Zn metabolism may be particularly affected. Zinc is essential for maintaining skin integrity because of its roles in the structure and function of DNA and RNA polymerases, cell proliferation, epidermal regeneration, and keratinization. Therefore, the decreased serum Zn concentrations observed in affected cattle may reflect increased physiological requirements for tissue repair and epithelial renewal, as well as enhanced metabolic utilization associated with the cellular immune response [16]. In addition, because Zn contributes to the structural stability and function of Cu/Zn-superoxide dismutase (Cu/Zn-SOD), reduced serum Zn concentrations may also be linked to the oxidative stress associated with dermatophytosis [36,37]. Consistent with this interpretation, serum Zn concentrations were positively correlated with CAT activity in the dermatophytosis group (r_s_ = 0.284, *p* < 0.05), suggesting that enzymatic antioxidant defense may be relatively better preserved in animals with more maintained Zn status. In this context, decreased serum Zn concentrations, together with reduced CAT activity and GSH levels and increased MDA levels, support a biochemical profile consistent with infection-associated redox imbalance in cattle with dermatophytosis. Taken together, these findings suggest that reduced serum Zn concentrations in dermatophytosis may reflect not only altered trace-element metabolism but also a systemic response associated with increased oxidative burden and impaired antioxidant defense.

The significantly lower serum Cu concentration observed in cattle with dermatophytosis than in healthy controls suggests that copper homeostasis is also affected in association with the disease. Copper is an essential cofactor for several enzymes, including lysyl oxidase, which is involved in collagen and elastin cross-linking and connective tissue stabilization, and tyrosinase, which plays a key role in melanin biosynthesis [38]. Therefore, the reduced serum Cu concentration detected in affected animals may reflect increased utilization related to tissue repair and inflammatory processes. During infection and inflammation, copper metabolism may be reorganized through changes in copper transport and tissue distribution; therefore, the reduced serum Cu concentration detected in affected animals may reflect altered copper utilization and redistribution related to inflammatory and tissue-repair processes [39]. In addition, copper has important structural and functional roles in antioxidant enzyme systems, particularly Cu/Zn-superoxide dismutase. Consistent with this biological framework, serum Cu concentration in the dermatophytosis group was positively correlated with CAT activity (r_s_ = 0.429, *p* < 0.01) and negatively correlated with MDA levels (r_s_ = −0.295, *p* < 0.05). These associations suggest that, in animals with relatively preserved Cu status, enzymatic antioxidant defense may be maintained more effectively, whereas lipid peroxidation may remain more limited. Taken together, the decreased serum Cu concentration observed in cattle with dermatophytosis may reflect altered copper utilization and distribution related to tissue repair and inflammation, as well as a redox-related response associated with reduced antioxidant capacity and increased lipid peroxidation.

In the present study, serum Se concentrations were significantly lower in cattle with dermatophytosis than in healthy controls, suggesting that selenium homeostasis may also be affected in association with the disease. Selenium plays an important role in maintaining cellular antioxidant defense and redox balance because it is incorporated into several selenoproteins, particularly members of the glutathione peroxidase (GPx) family [40]. In the context of dermatophyte-associated inflammatory responses and ROS-mediated oxidative processes, the decreased serum Se concentrations observed in cattle with dermatophytosis may be compatible with the increased oxidative burden associated with the disease [23,24]. Within this framework, the decreased serum Se concentrations observed in cattle with dermatophytosis may be consistent with the increased oxidative burden associated with the disease. However, no statistically significant correlations were detected between serum Se concentrations and CAT activity, GSH levels, or MDA levels in the present study (Table 4). This finding suggests that the contribution of Se to redox balance may not have been directly reflected by the oxidative stress/antioxidant defense parameters measured here, and may instead be mediated predominantly through other selenoprotein-dependent mechanisms, particularly the glutathione peroxidase system. Thus, although the decrease in serum Se concentrations is compatible with an oxidative stress-related response, a direct association between Se status and the redox markers evaluated in this study could not be demonstrated.

In the present study, cattle with dermatophytosis showed significantly lower serum CAT activity and GSH levels, together with significantly higher MDA levels, suggesting a shift toward oxidative stress and weakened antioxidant defense. CAT, GSH, and MDA collectively reflect enzymatic antioxidant defense, non-enzymatic redox buffering, and lipid peroxidation, respectively [24,25,26]. Therefore, the observed pattern of decreased CAT activity and GSH levels alongside increased MDA levels is consistent with increased oxidative burden and impaired antioxidant capacity in cattle with dermatophytosis. In addition, the negative correlation between MDA and GSH levels in the dermatophytosis group (r_s_ = −0.305, *p* < 0.05) suggests that increased lipid peroxidation may be accompanied by depletion of antioxidant reserves. Consistent with these findings, a recent study in cattle with trichophytosis also reported increased MDA levels together with decreased CAT activity and other antioxidant defense markers, supporting the view that the disease may be associated with biochemical alterations related to oxidative stress [27]. From this perspective, the coexistence of decreased CAT activity and GSH levels with increased MDA levels in the present study suggests that dermatophytosis may be associated not only with superficial cutaneous lesions but also with a systemic biochemical stress response affecting host redox homeostasis. The selection of CAT, GSH, and MDA in the present study was intended to provide a focused evaluation of enzymatic antioxidant defense, non-enzymatic antioxidant capacity, and lipid peroxidation, respectively. However, these markers do not fully characterize the systemic physiological response to dermatophytosis. Additional biomarkers, including other antioxidant enzymes and inflammatory or acute-phase indicators such as C-reactive protein, haptoglobin, or serum amyloid A, could provide a more comprehensive assessment of the relationship between oxidative stress, inflammation, and serum biochemical alterations in affected cattle. Therefore, the redox findings of the present study should be interpreted as part of a limited biomarker panel rather than as a complete representation of the systemic response to infection.

The separate evaluation of correlation patterns in the dermatophytosis and healthy control groups suggests that the relationship between mineral status and redox balance was not limited to between-group differences, but also differed according to disease status. In the dermatophytosis group, the significant correlations of certain serum macro- and trace-element concentrations with CAT activity and MDA levels, together with the negative correlation between Co and GSH levels, indicate that the interaction between mineral homeostasis and oxidative stress/antioxidant defense responses may become more evident during disease. By contrast, in the healthy control group, these relationships were largely absent, except for the positive correlation between CAT activity and Ca concentration, suggesting that this biochemical interplay may be more limited under healthy conditions. Overall, these correlation patterns support the interpretation that bovine dermatophytosis may be associated with a systemic biochemical response in which mineral homeostasis and redox balance are interrelated.

A similar pattern was also observed for iron metabolism. In the present study, serum Fe concentrations were significantly lower in cattle with dermatophytosis than in healthy controls, suggesting that iron homeostasis may be affected in association with the disease. This finding may be interpreted within the framework of nutritional immunity, a host defense response that develops during infection to restrict pathogen access to essential nutrients. Pathogenic microorganisms, including *Trichophyton* spp. require iron to support their proliferation and virulence [41]. During infection, the host may reduce extracellular iron availability through iron sequestration and hepcidin–ferroportin-mediated regulation of iron export [42]. Accordingly, the decreased serum Fe concentrations observed in cattle with dermatophytosis may reflect a protective metabolic response to infection rather than simple mineral deficiency alone. Nevertheless, the positive correlation between serum Fe concentrations and CAT activity (r_s_ = 0.359, *p* < 0.05), together with the negative correlation between serum Fe concentrations and MDA levels (r_s_ = −0.294, *p* < 0.05), suggests that iron homeostasis may also be linked to redox balance in affected animals. These findings indicate that reduced serum Fe concentrations may represent, on the one hand, a component of the host defense response and, on the other hand, a biochemical alteration associated with weaker antioxidant defense and increased lipid peroxidation within the dermatophytosis group.

The significantly lower serum Mg, Ca, and Na concentrations observed in cattle with dermatophytosis than in healthy controls suggest that the disease may be associated not only with changes in trace-element status but also with alterations in macromineral homeostasis. Several physiological and behavioral mechanisms may contribute to these changes. Although pruritus in bovine dermatophytosis is variable, irritation and discomfort associated with skin lesions may contribute to reduced feed intake and stress-related metabolic responses [7,15]. In ruminants, serum macromineral concentrations are strongly influenced by dietary intake, gastrointestinal absorption, mineral interactions, and homeostatic regulatory mechanisms [16,43]. In addition, stress-related alterations in gastrointestinal barrier integrity and digestive and absorptive functions may compromise mineral bioavailability and intestinal absorption [43,44,45]. Accordingly, the decreased serum Mg, Ca, and Na concentrations observed in the present study may reflect the combined effects of reduced feed intake, altered gastrointestinal function, and disease-associated metabolic adaptation on macromineral homeostasis. Nevertheless, in the dermatophytosis group, serum Mg concentration was positively correlated with CAT activity (r_s_ = 0.398, *p* < 0.01) and negatively correlated with MDA levels (r_s_ = −0.314, *p* < 0.05), suggesting that Mg homeostasis may also be linked to redox balance. Likewise, the positive correlation between serum Na concentration and CAT activity (r_s_ = 0.450, *p* < 0.01) suggests that alterations in sodium balance may not be entirely independent of the antioxidant response.

Another possible mechanism underlying the alterations in macromineral concentrations may involve changes in serum protein binding associated with the metabolic response to infection. During infectious and inflammatory processes, serum albumin may decrease as part of the systemic inflammatory and metabolic response, and hypoalbuminemia is clinically relevant because albumin is a major binding protein for several circulating molecules and minerals [46,47]. Although serum albumin concentrations were not measured in the present study, approximately half of circulating calcium is bound to serum proteins, mainly albumin; similarly, approximately one-third of plasma magnesium is protein-bound, with albumin representing the major binding protein for this fraction [48,49]. Therefore, disease-associated alterations in protein metabolism may have contributed to the lower total serum Ca and Mg concentrations observed in cattle with dermatophytosis. By contrast, the absence of significant correlations between Ca concentration and CAT activity, GSH levels, or MDA levels in the dermatophytosis group suggests that the decrease in total serum Ca may be more closely related to protein-binding dynamics and broader homeostatic disturbances than to the redox markers evaluated in this study. Taken together, the macromineral alterations identified in the present study may have resulted from the combined influence of reduced feed intake, stress-related metabolic responses, possible changes in gastrointestinal absorption, and infection-associated metabolic adaptation.

One of the most noteworthy findings of the present study was the significantly higher serum Co and Cr concentrations observed in cattle with dermatophytosis than in healthy controls (Table 2). This pattern differs from the decreases observed for several other macro and trace elements in the affected group and suggests that trace elements may not respond uniformly during dermatophytosis. Trace element profiles may change during systemic inflammatory and stress-related metabolic states, and such alterations are often element-specific rather than uniform [50]. Accordingly, the increased Co and Cr concentrations observed in the present study may reflect element-specific alterations in serum trace-element distribution associated with the host response to dermatophytosis.

In the present study, serum Cr concentrations were significantly higher in cattle with dermatophytosis than in healthy controls (Table 2), suggesting that cr homeostasis may also be affected in association with the disease. Cr has been associated with insulin action and carbohydrate metabolism, and its biological relevance in cattle has also been discussed in relation to stress-related metabolic regulation [51]. Cr has also been discussed in relation to stress-associated immune and metabolic regulation in cattle, although responses may vary depending on physiological and experimental conditions [17]. Therefore, the elevated serum Cr concentrations detected in the present study should not be attributed to a single definitive mechanism; rather, they may represent an element-specific alteration in serum distribution accompanying stress-related metabolic reorganization during dermatophytosis. Consistent with this interpretation, the positive correlation between serum Cr concentration and CAT activity in the dermatophytosis group (r_s_ = 0.314, *p* < 0.05; Table 4) suggests that Cr alterations may not be entirely independent of the antioxidant response. Nevertheless, the absence of significant correlations between Cr concentration and either GSH or MDA indicates that this finding should be interpreted cautiously.

Similarly, serum Co concentrations were significantly higher in cattle with dermatophytosis than in healthy controls (Table 2), suggesting that cobalt homeostasis may also be affected in association with the disease. Because cobalt is a structural component of vitamin B12, it has biological importance in ruminants for ruminal microbial activity, propionate metabolism, and energy homeostasis [22,52]. In contrast to monogastric species, Co metabolism in ruminants is closely linked to rumen microorganisms and vitamin B12 synthesis; therefore, disease-associated changes in appetite, ruminal activity, and metabolic status may influence both the biological utilization and circulating concentration of this element [52,53]. In this context, the negative correlation between serum Co concentrations and GSH levels in the dermatophytosis group (r_s_ = −0.290, *p* < 0.05; Table 4) suggests that increased Co concentrations may be associated with a disease-related metabolic redistribution pattern distinct from those of trace elements directly involved in antioxidant defense. However, the absence of significant correlations between Co concentration and either CAT activity or MDA levels limits the interpretation of this finding in relation to direct oxidative damage markers. Therefore, the increase in serum Co concentrations observed in the present study may be regarded as an element-specific biochemical alteration accompanying changes in ruminal function, energy metabolism, and disease-associated metabolic adaptation during dermatophytosis. Notably, a recent report showing higher serum Co concentrations in young Holstein cattle with natural trichophytosis than in healthy controls, followed by a partial decrease after vaccination, is also consistent with this interpretation [54].

## 5. Conclusions

This study showed that bovine dermatophytosis was predominantly associated with *Trichophyton verrucosum* and that the disease may not be limited to superficial skin lesions, but may also be accompanied by systemic biochemical alterations related to mineral homeostasis and redox balance. In cattle with dermatophytosis, serum Cu, Zn, Se, Mg, Ca, Na, and Fe concentrations were lower, whereas Co and Cr concentrations were higher than those in healthy controls. These changes, together with decreased CAT activity and GSH levels and increased MDA levels, suggest that both the macro- and trace-element profile and oxidative stress/antioxidant defense balance may be affected in association with dermatophytosis. In addition, within-group correlation analyses, particularly the associations observed between selected mineral concentrations and redox markers in cattle with dermatophytosis, suggest that these biochemical relationships may become more evident under disease conditions. However, because this study was observational in nature, no definitive conclusions can be drawn regarding the causal mechanisms or clinical consequences of the detected alterations. Overall, the combined evaluation of mineral profile alterations and oxidative stress/antioxidant defense parameters in bovine dermatophytosis may contribute to a more comprehensive understanding of the pathobiology of the disease; nevertheless, further controlled studies are required to clarify the mechanistic basis and clinical significance of these relationships.

Although this study provides original data through the combined evaluation of serum macro- and trace-element concentrations and oxidative stress/antioxidant defense parameters in bovine dermatophytosis, particularly by documenting increased Co and Cr concentrations, several limitations should be considered when interpreting the findings.

First, because the study was observational and conducted under field conditions, the biochemical differences observed between the dermatophytosis and healthy control groups cannot be attributed solely to dermatophytosis. Although group, sex, and age were considered in the revised statistical analysis, residual variability related to breed type, farm-level management, feeding practices, environmental conditions, and individual disease characteristics cannot be completely excluded. Both groups consisted of calves and young cattle aged 2–12 months, and adult lactating cows, pregnant cows, feedlot-finishing steers, and mature breeding bulls were not included; however, the animals were not stratified according to breed type or narrower age subgroups. In addition, although blood samples from both groups were collected during the same seasonal study period, between February and May 2026, and during routine daytime working hours, approximately between 09:00 and 17:00, exact individual sampling times were not recorded as a separate variable. Moreover, although lesion distribution was recorded during clinical examination, a predefined published or validated dermatophytosis severity scoring system was not applied prospectively, and lesion number or lesion extent was not recorded in a standardized quantitative manner for all affected animals; therefore, severity-related effects on serum mineral and redox parameters could not be assessed. The control animals were selected from the same geographical region and broadly similar field management conditions; however, they were not individually matched with affected cattle at the farm level, and equal numbers of dermatophytosis and control animals from each farm could not be achieved under field conditions. Therefore, possible farm-related and management-related influences should be considered when interpreting the findings.

Consequently, despite the inclusion of age and sex in the revised statistical analysis, residual variability related to breed type, physiological status, exact sampling time, diurnal variation, infection severity, and farm-level management may have contributed to part of the variation observed between groups, even though broadly similar regional management, housing, and feeding conditions were taken into account.

Another limitation is that dermatophyte identification was based on conventional mycological methods, including direct microscopic examination, culture characteristics, colony morphology, and microscopic morphology. Although these methods are useful for routine species-level identification, they may not fully exclude misidentification among closely related dermatophytes or allow evaluation of strain-level, variant, or subspecies-level differences. Such pathogen-related variability could potentially influence lesion severity, host response, and the measured serum mineral and redox parameters. Therefore, future studies using PCR-based identification, sequencing, or molecular typing approaches may provide more accurate and detailed information on dermatophyte diversity and its possible relationship with systemic biochemical alterations.

In addition, serum albumin, ceruloplasmin, cortisol, additional antioxidant enzymes and inflammatory or acute-phase biomarkers such as C-reactive protein, haptoglobin, and serum amyloid A were not evaluated in this study, although these parameters could have provided more comprehensive information on the physiological, inflammatory, and redox-related responses associated with dermatophytosis.

Therefore, although the decreases in Cu, Zn, and Se concentrations and the increase in Cr concentration can be discussed in relation to the acute-phase response, oxidative stress, and endocrine/metabolic responses in light of the literature, these findings should not be interpreted causally. In addition, rumen content, liver tissue, and vitamin B12-related metabolic parameters were not examined, limiting a clearer interpretation of the biological basis of the increased Co and Cr concentrations, which were among the notable findings of this study.

Finally, dietary and environmental variation should be considered an important limitation of the present study. Although the animals were selected from farms within the same region and were maintained under a broadly similar traditional feeding system based mainly on summer-harvested meadow hay offered together with straw during winter, the diets of the control and dermatophytosis groups were not identical. In particular, differences in pasture origin, hay and straw quality, mineral supplementation practices, water mineral content, and soil mineral profiles were not systematically recorded or chemically analyzed. Therefore, a detailed nutrient-content comparison between groups could not be performed, and it is not possible to fully exclude dietary or environmental contributions to the serum macro- and trace-element differences observed between groups. For this reason, the mineral alterations detected in this study should be interpreted as associations observed under field conditions and not as changes attributable solely to dermatophytosis. Within this framework, future studies may benefit from evaluating hair samples in addition to serum as a complementary biomonitoring matrix to identify environmental element exposure and longer-term patterns of mineral accumulation [55]. Accordingly, future controlled, multicenter studies, preferably supported by detailed ration records, chemical analyses of feed, water, and soil, and tissue-level and/or molecular analyses, are needed to more clearly elucidate the mechanistic basis and clinical significance of the biochemical alterations associated with dermatophytosis.

## Figures and Tables

**Table 1:** Numerical and percentage distribution of dermatophyte species isolated from cattle with dermatophytosis.

Dermatophyte Species	Number of Isolates (n)	Percentage (%)
*Trichophyton verrucosum*	45	90
*Trichophyton mentagrophytes*	5	10
Total	50	100

**Table 2:** Comparison of serum macro- and trace-element concentrations between healthy control cattle and cattle with dermatophytosis.

Parameter	Control (LSM ± SE)	Dermatophytosis (LSM ± SE)	*p* Value
Cu (µg/mL)	1.016 ± 0.031	0.866 ± 0.022	*p* < 0.01
Zn (µg/mL)	2.202 ± 0.065	1.839 ± 0.046	*p* < 0.01
Co (µg/mL)	0.126 ± 0.004	0.143 ± 0.003	*p* < 0.01
Se (µg/mL)	0.141 ± 0.004	0.116 ± 0.003	*p* < 0.01
Mo (µg/mL)	0.557 ± 0.030	0.582 ± 0.021	*p* = 0.500
Mn (µg/mL)	1.279 ± 0.060	1.314 ± 0.042	*p* = 0.638
Mg (µg/mL)	39.08 ± 1.583	33.885 ± 1.116	*p* = 0.009
Ca (µg/mL)	100.002 ± 1.519	81.605 ± 1.071	*p* < 0.001
Na (µg/µL)	3.357 ± 0.128	2.875 ± 0.090	*p* < 0.01
Fe (µg/mL)	6.195 ± 0.196	5.245 ± 0.138	*p* < 0.01
Cr (µg/mL)	0.525 ± 0.014	0.602 ± 0.010	*p* < 0.01

Data are presented as LSM ± SE (Least Square Mean ± Standard error). Na is expressed as µg/µL; all other elements are expressed as µg/mL. *p* < 0.05 was considered statistically significant.

**Table 3:** Comparison of serum oxidative stress and antioxidant defense parameters between healthy control cattle and cattle with dermatophytosis.

Parameter	Control (LSM ± SE)	Dermatophytosis (LSM ± SE)	*p* Value
CAT (I/U)	18.90 ± 1.36	4.67 ± 0.74	*p* < 0.001
GSH (µmol/L)	1.71 ± 0.051	0.716 ± 0.036	*p* < 0.001
MDA (µmol/L)	8.63 ± 2.43	38.98 ± 1.75	*p* < 0.001

Data are presented as LSM ± SE (Least Square Mean ± Standard error). CAT, catalase; GSH, reduced glutathione; MDA, malondialdehyde. *p* < 0.05 was considered statistically significant.

**Table 4:** Spearman correlation coefficients among serum macro- and trace-element levels and oxidative stress/antioxidant defense parameters in cattle with dermatophytosis.

Variable	Cu (µg/mL)	Zn (µg/mL)	Co (µg/mL)	Se (µg/mL)	Mo (µg/mL)	Mn (µg/mL)	Mg (µg/mL)	Ca (µg/mL)	Na (µg/µL)	Fe (µg/mL)	Cr (µg/mL)	CAT (I/U)	GSH (µmol/L)
Zn (µg/mL)	0.756 **												
Co (µg/mL)	0.563 **	0.734 **											
Se (µg/mL)	0.714 **	0.972 **	0.712 **										
Mo (µg/mL)	0.695 **	0.327 *	0.263	0.352 *									
Mn (µg/mL)	0.821 **	0.579 **	0.380 **	0.598 **	0.935 **								
Mg (µg/mL)	0.908 **	0.745 **	0.409 **	0.689 **	0.624 **	0.801 **							
Ca (µg/mL)	0.316 *	0.406 **	0.499 **	0.380 **	0.102	0.154	0.265						
Na (µg/µL)	0.895 **	0.856 **	0.507 **	0.865 **	0.566 **	0.771 **	0.876 **	0.221					
Fe (µg/mL)	0.964 **	0.811 **	0.596 **	0.772 **	0.700 **	0.848 **	0.902 **	0.326 *	0.899 **				
Cr (µg/mL)	0.884 **	0.793 **	0.760 **	0.753 **	0.551 **	0.677 **	0.759 **	0.537 **	0.785 **	0.884 **			
CAT (I/U)	0.429 **	0.284 *	0.207	0.254	0.268	0.349 *	0.398 **	0.165	0.450 **	0.359 *	0.314 *		
GSH (µmol/L)	−0.145	−0.148	−0.290 *	−0.108	−0.022	−0.047	−0.155	−0.176	−0.157	−0.119	−0.243	−0.103	
MDA (µmol/L)	−0.295 *	−0.052	0.072	−0.038	−0.413 **	−0.385 **	−0.314 *	0.179	−0.176	−0.294 *	−0.111	−0.242	−0.305 *

Values represent Spearman’s rank correlation coefficients. * *p* < 0.05; ** *p* < 0.01.

**Table 5:** Spearman correlation coefficients among serum macro- and trace-element concentrations and oxidative stress/antioxidant defense parameters in healthy control cattle.

Variable	Cu (µg/mL)	Zn (µg/mL)	Co (µg/mL)	Se (µg/mL)	Mo (µg/mL)	Mn (µg/mL)	Mg (µg/mL)	Ca (µg/mL)	Na (µg/µL)	Fe (µg/mL)	Cr (µg/mL)	CAT (I/U)	GSH (µmol/L)
Zn (µg/mL)	0.761 **												
Co (µg/mL)	0.442 *	0.696 **											
Se (µg/mL)	0.752 **	0.989 **	0.699 **										
Mo (µg/mL)	0.813 **	0.390	0.236	0.377									
Mn (µg/mL)	0.919 **	0.579 **	0.333	0.567 **	0.962 **								
Mg (µg/mL)	0.939 **	0.685 **	0.273	0.674 **	0.767 **	0.880 **							
Ca (µg/mL)	0.426 *	0.582 **	0.331	0.582 **	0.265	0.326	0.385						
Na (µg/µL)	0.972 **	0.826 **	0.457 *	0.818 **	0.713 **	0.861 **	0.953 **	0.375					
Fe (µg/mL)	0.966 **	0.761 **	0.442 *	0.752 **	0.813 **	0.919 **	0.939 **	0.426 *	0.972 **				
Cr (µg/mL)	0.934 **	0.857 **	0.558 **	0.852 **	0.672 **	0.797 **	0.813 **	0.554 **	0.903 **	0.933 **			
CAT (I/U)	0.101	0.298	−0.167	0.298	0.065	0.096	0.123	0.653 **	0.075	0.101	0.232		
GSH (µmol/L)	−0.263	−0.369	−0.362	−0.367	−0.160	−0.202	−0.279	−0.157	−0.328	−0.263	−0.233	0.360	
MDA (µmol/L)	0.219	−0.024	−0.013	−0.026	0.208	0.214	0.143	−0.145	0.163	0.219	0.144	−0.091	0.009

Values represent Spearman’s rank correlation coefficients. * *p* < 0.05; ** *p* < 0.01.

## Data Availability

The original contributions presented in this study are included in the article. Further inquiries can be directed to the corresponding author.

## Abbreviations

Ca	Calcium
CAT	Catalase
Co	Cobalt
Cr	Chromium
Cu/Zn-SOD	Copper/zinc superoxide dismutase
Cu	Copper
DNA	Deoxyribonucleic acid
DTM	Dermatophyte Test Medium
DTNB	5,5′-dithiobis-(2-nitrobenzoic acid)
Fe	Iron
GLM	General linear model
GPx	Glutathione peroxidase
GSH	Reduced glutathione
ICP-OES	Inductively coupled plasma optical emission spectroscopy
KOH	Potassium hydroxide
LPCB	Lactophenol cotton blue
LSM	Least-squares mean
MDA	Malondialdehyde
Mg	Magnesium
Mn	Manganese
Mo	Molybdenum
Na	Sodium
RNA	Ribonucleic acid
ROS	Reactive oxygen species
Se	Selenium
SE	Standard error
SOD	Superoxide dismutase
SPSS	Statistical Package for the Social Sciences
Zn	Zinc
